# Control effect of dual-mechanism myopia control lenses on myopia in children and adolescents: a prospective, 12-month, non-randomized, parallel-controlled, and single-center trial protocol

**DOI:** 10.3389/fmed.2026.1931165

**Published:** 2026-09-10

**Authors:** Ling Wang, Qing Zhang, Shao-qi Zhou, Jun Huang, Ting-Ting Zhou, Yue-Si Gu, Wen-xin Wang

**Affiliations:** 1 Department of Optometry Engineering, Jinling Institute of Technology, Nanjing, China; 2 Ophthalmology Department, Nanjing Vision Jingling Eye Hospital, Nanjing, China; 3 Ophthalmology Department, Fuyang Vision Zeming Eye Hospital, Fuyang, China

**Keywords:** children, clinical trial, efficacy, myopia, peripheral defocus, retinal contrast-Designed lenses (RCDL), safety, spectacle

## Abstract

**Background:**

Myopia has become an increasingly serious global issue. Preventing myopia and avoiding the risks of high myopia has become an important concern for children, adolescents and their parents. Dual-mechanism myopia control lenses (DMMCL) is a specialized design spectacle lens based on peripheral defocus theory and retinal contrast theory. Although the benefits of peripheral defocus lenses and retinal contrast-designed lenses (RCDL) in myopia prevention have been confirmed, whether the lenses integrating both two designs can effectively prevent further progression of myopia still requires further research.

**Purpose:**

To report the protocol of a clinical trial designed to evaluate the efficacy and safety of DMMCL vs. RCDL alone for slowing the progression of myopia among school-age children in China. The primary objective of this study is to evaluate whether DMMCL are equivalent to RCDL in controlling myopia progression.

**Method and analysis:**

This is a prospective, single-center, non-randomized, equivalence trial designed to compare the efficacy and safety of DMMCL vs. RCDL for controlling myopia progression in children aged 6–12 years over a 12-month period. The primary outcome is change in axial length (AL) from baseline to 12 months. We hypothesize that the two treatments are equivalent within a prespecified margin of ±0.15 mm. This study will enroll 120 myopic children, aged 6–12 years, with cycloplegic spherical equivalent (SE) ranging from −0.50 D to −6.00 D in both eyes. Eligible participants will be assigned to the DMMCL group (*n* = 60) or the RCDL group (*n* = 60) according to their own preferences, or those of their guardians. Participants will be advised to wear their assigned spectacles full-time throughout the trial. All participants will be advised to engage in at least 2 h outdoors per day and will be scheduled for regular follow-up visits. AL, cycloplegic SE, and binocular visual function will be assessed at baseline and throughout the 12-month study period. Both groups will complete questionnaires on adaptation and visual quality one week and one month after wearing the lenses. Participants from both groups will require a follow up visit every 3 months. The primary endpoint is the 12-month change from baseline in AL. Secondary outcomes include changes in cycloplegic SE and binocular vision. Adaption and vision quality questionnaire results are pre-specified outcomes.

**Outcome:**

Primary outcomes: Twelve-month changes in AL Secondary outcomes: Twelve-month changes in SE from baseline and binocular visual function parameters. Other pre-specified outcomes Adaption and vision quality questionnaire results.

**Conclusion:**

The future findings of this trial are expected to provide valuable reference for optimizing myopia management strategies and may contribute to the evidence base that informs pediatric eye care policy.

**Trial Registration**: https://www.chictr.org.cn/, https://www.chictr.org.cn/showproj.html?proj=301629, identifier ChiCTR2600116438.

## 1 Backgroud

Myopia has become an increasingly serious global issue. Over the past decades, childhood myopia has emerged as a leading public health challenge in East Asia (1–3). Despite a plateau in overall myopia prevalence, the rising tide of high myopia necessitates targeted intervention strategies. Although overall myopia prevalence appears to have stabilized in recent years, the persistently rising proportion of high myopia underscores the urgent need for more targeted preventive strategies (3). Previous studies have shown that myopia onset during early adolescence is associated with a higher risk of rapid progression to high myopia in adulthood (4). High myopia and its complications may place substantial strain on healthcare systems and health insurance programs. Therefore, there is an urgent need for therapeutic strategies that can effectively delay myopia progression and thereby reduce the long-term risk of high myopia. Preventing myopia and avoiding the risks of high myopia has become an important concern for children, adolescents and their parents. Numerous treatment approaches (pharmacological, behavioral, environmental, and optical) have been proposed to decelerate the progression of myopia, particularly in pediatric populations. Among these, spectacle lenses represent a straightforward and less intrusive therapeutic approach for children and their parents (5). Recent years have witnessed the development of various spectacle lens technologies aimed at slowing myopia progression and enhancing the quality of life of affected children, potentially alleviating the associated long-term healthcare burden.

Currently, common specially designed spectacle lenses primarily include defocus incorporated multiple segments (DIMS) (6, 7) lens based on peripheral defocus theory, highly aspherical lenslets (HAL) (8, 9) lenses, and others. efforts have been around designing spectacles that optically reduce or eliminate hyperopic defocus in the periphery. They also encompass cylindrical annular refractive element (CARE) lenses based on higher-order aberration theory (10, 11), as well as diffusion optics technology (DOT) spectacle lenses incorporating retinal contrast design theory (12, 13).

Among various myopia control strategies, spectacle lenses designed for myopia control are considered the least invasive option and offer a favorable safety profile for long-term use (2, 14). Spectacle lenses incorporating technologies such as DIMS (6), HAL (15), DOT (12), and CARE lenses have been shown to exhibit a reduction in refractive progression and axial elongation in comparison to single-vision lenses (16). Recent research shows that DOT lenses provide a visual experience that is clinically equivalent to standard single vision lenses (17). Research's findings lend support to the use of specialty lenses as an effective, non-invasive strategy to reduce the risks associated with high myopia and to guide optimal lens selection in clinical practice (18).

Spectacles designed based on the sole peripheral defocus theory (15, 19) and sole retinal contrast theory have demonstrated significant myopia control efficacy (13). However, whether integrating both peripheral defocus and retinal contrast design principles within a single lens can achieve superior control requires evidence-based medical investigation.

DMMCL is a specialized design spectacle lens based on peripheral defocus theory and retinal contrast theory. The primary objective of this study is to evaluate whether DMMCL are equivalent to RCDL in controlling myopia progression, as measured by AL elongation over 12 months. We hypothesize that the difference in AL change between the two groups will fall within the prespecified equivalence margin of ±0.15 mm, indicating clinically meaningful equivalence.

This study also aims to characterize the design features of the DMMCL, evaluate its myopia control efficacy, and examine its clinical applicability, with particular reference to its dual-mechanism design integrating peripheral defocus and retinal contrast modulation.

## 2 Material and methods

### 2.1 Study design

To evaluate the effectiveness and safety of DMMCL among myopic children and adolescent, we propose to conduct a prospective, non-randomized, parallel-controlled, single center clinical trial. This trial has been approved by Nanjing Vision Jingling Eye Hospital Institutional Review Board (No. 2025-12-001-k001) and registered at at https://www.chictr.org.cn/ as chiCTR2600116438. Written informed consent will be obtained from the parents or legal guardians of all participating children, and assent will be sought from the children themselves where applicable. The study protocol was designed and will be executed in compliance with the ethical principles of the Declaration of Helsinki, as well as applicable national regulations.

This study is a prospective, non-randomized, active-controlled, equivalence trial. Participants will be assigned in a 1:1 ratio to receive either DMMCL group or RCDL group according to their own preferences, or those of their guardians. The equivalence margin for the primary outcome (AL change over 12 months) was set at ±0.15 mm, based on the established efficacy difference between DOT and single-vision lenses reported in the CATHAY study (20) (−0.26 mm for axial length at 12 months). An equivalence margin of ±0.15 mm ensures that the DOT lens retains at least 55% of the clinically meaningful effect observed against single-vision lenses, while accommodating the inherent variability across studies and populations.

The trial schedule followed the SPIRIT (Standardized Protocol Items: Recommendations for Interventional Trials) 2025 statement (https://www.consort-spirit.org) (21).

### 2.2 Participants' recruitment and eligibility

Study participants will be recruited over a twelve-month period from January to December 2026. To facilitate participant recruitment, a poster advertisement will be displayed at the hospital. A clinical research coordinator (CRC) will initially contact potentially eligible candidates and their parents or legal guardians to provide detailed information about the trial, including its purpose, expected duration, and participant obligations. Written informed consent will then be obtained from both the candidates and their parents/guardians. Following consent, candidates will undergo a screening evaluation. Those who meet all inclusion criteria and none of the exclusion criteria will be formally enrolled in the trial.

Inclusion criteria were as follows:

1. Children aged 6–12 years at enrollment, with no restriction on sex;
2. Those with normal eye examinations outcomes;
3. Cycloplegic optometry examination (Both eyes: −0.50D to −6.00D; Astigmatism ≤1.50 D and anisometropia ≤1.50 D in both eyes.)
4. Best-corrected distance visual acuity (BCDVA) of 1.0 (decimal equivalent) or better in each eye, as measured under standardized conditions;
5. Be able to participate in follow-up visits.

Exclusion criteria were as follows:

1. A history of ocular trauma or intraocular surgery;
2. Strabismus or any ocular motility disorder
3. Have used or have previously used other myopia control methods in three months
4. Poor compliance, unable to follow up at the prescribed time.

### 2.3 Randomization and masking

Participants will be allocated in a 1:1 ratio to either the intervention group or the control group based on their preference, rather than by randomization.

Owing to the inherent nature of the intervention. The surface appearance of lenses of different designs varies considerably, blinding of participants and the principal investigator was not feasible; therefore, this was an open-label trial for these parties. However, outcome assessors including technicians and optometrists, will be masked to treatment allocation.

### 2.4 Intervention

Intervention measures include wearing the DMMCL and RCDL all day, along with maintaining routine care such as recommending 120 min of daily outdoor activity. At each follow-up visit, study staff will reinforce importance of increased outdoor time for myopia control to all participants. We will urge them to ensure that their daily consistent wear time is no less than 12 h. Adaption and vision quality questionnaire results will be recorded in one week and one month after wearing the lenses.

**Figure 1:**
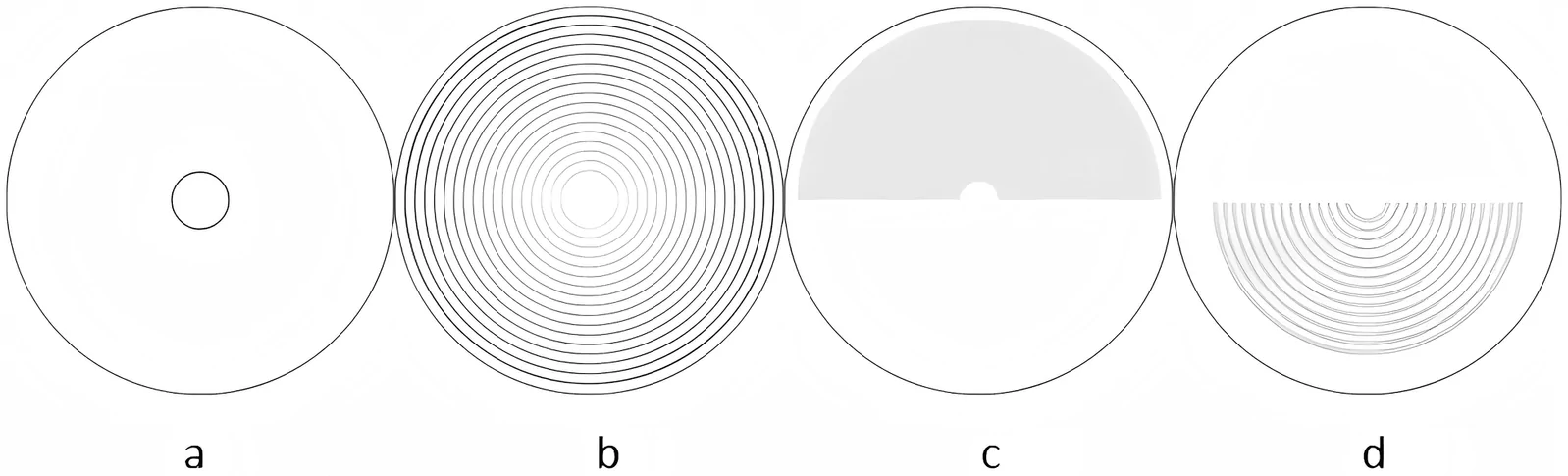
Basic design principles of dual-mechanism myopia control lenses (DMMCL) **(a)** central clear vision zone measures 8.50 mm **(b)** the back surface features a fully rotationally symmetric design based on the principle of peripheral defocus **(c)** half of the entire back surface is designed with a multi-point spiral haze structure based on retinal contrast principle **(d)** half of the entire front surface is designed with a concentric ring asymmetric multi-point micro-convex design based on the principle of peripheral defocus.

DMMCL is a specially designed lens. Its central clear vision zone measures 8.50 mm, ensuring normal distance vision and visual field requirements. The back surface features a fully rotationally symmetric design based on the principle of peripheral defocus to reduce peripheral hyperopic defocus. Additionally, half of the entire back surface is designed with a multi-point spiral haze structure based on retinal contrast principle, while half of the entire front surface is designed with a concentric ring asymmetric multi-point micro-convex design based on the principle of peripheral defocus (Figure 1).

DMMCL (Treatment group, EASSILLA, JiangSu, china) and RCDL [Control group, Diffusion Optics Technology (DOT), California, USA] are custom-fabricated plastic spectacle lenses. Participants will be instructed to wear the assigned spectacle lenses during waking hours, except during sleep and showering, throughout the 12-month trial period. Especially when doing near work. Daily wear time shall be no less than 12 h.

If a myopic shift of ≥0.50 D in SE is detected in either eye, participants will be refitted with the updated study spectacles. At the time of lens dispensing and at each subsequent follow-up visit, standardized verbal and written instructions regarding spectacle maintenance and daily wear requirements will be provided to both participants and their parents or guardians by trained study personnel. Use of topical atropine (any concentration) will be prohibited in both groups throughout the study period. At each scheduled 3-month follow-up visit, participants will receive standardized counseling on adherence to the prescribed eye care regimen, including the importance of maintaining 120 min of daily outdoor time.

**Figure 2:**
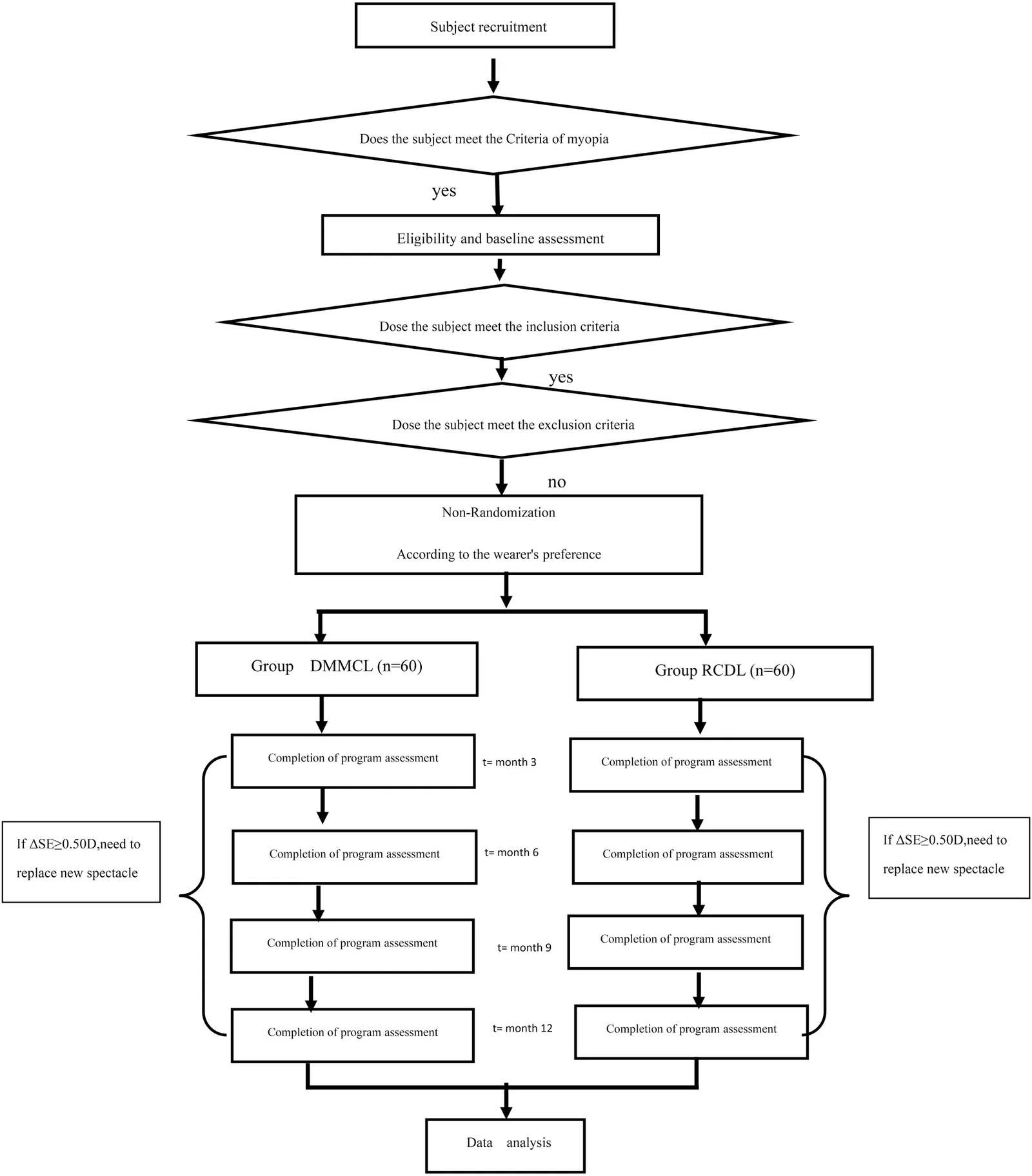
Flow diagram of study design.

At each scheduled visit, the study will evaluate AL, cycloplegic SE, visual acuity (VA), and binocular visual function in all participants. Adverse events reported by participants or their parents/guardians will be documented throughout the study period. The flow diagram of study design is shown in Figure 2.

### 2.5 Outcomes and follow-up schedule

**Figure 3:**
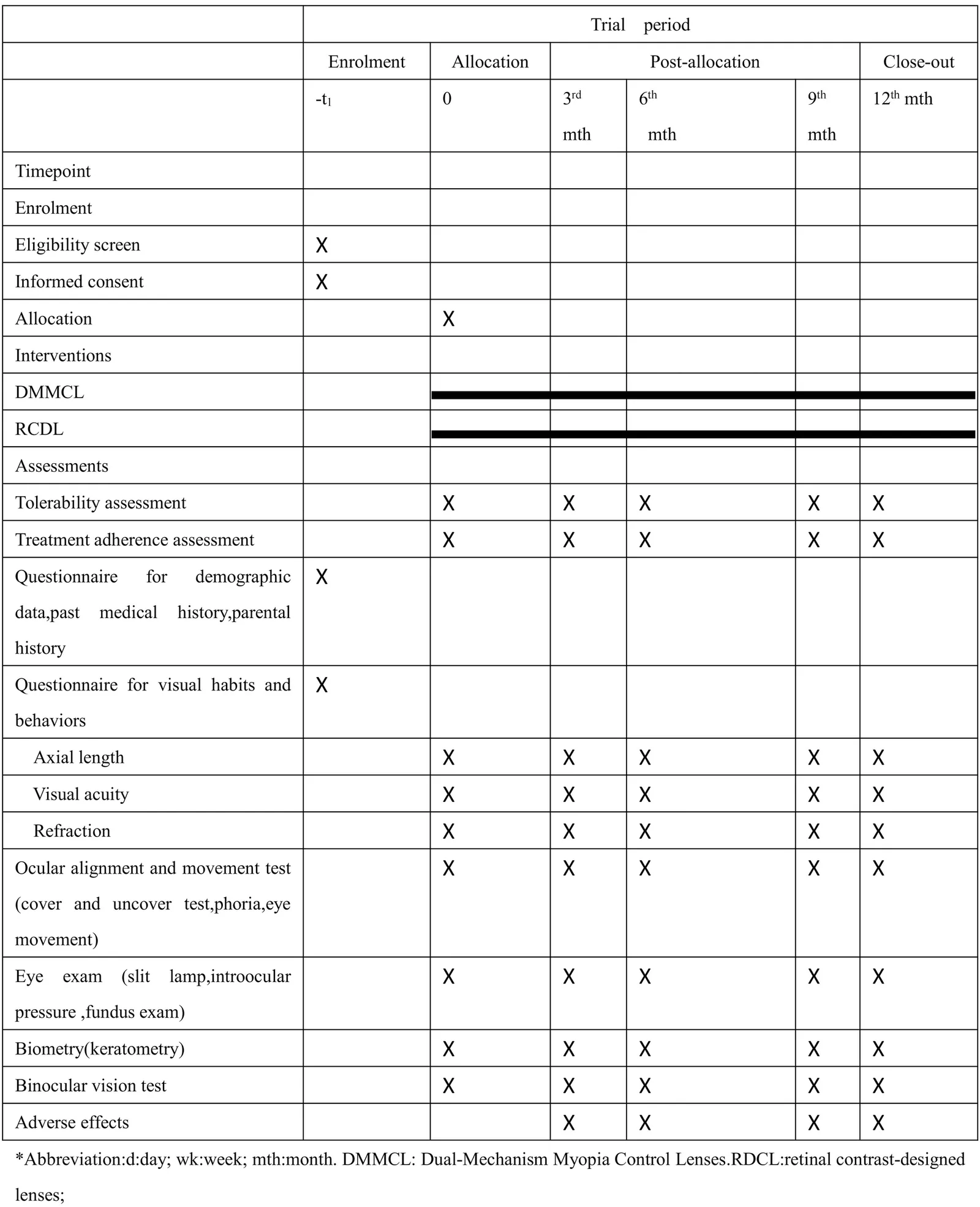
Schedule of assessment and examination items.

Cycloplegic SE and AL are widely regarded as the most relevant and reliable indicators for monitoring refractive progression in children and adolescents. Additionally, several factors are associated with myopic shift of SE. Accordingly, the primary, secondary, and exploratory outcomes will be assessed at each scheduled follow-up visit according to the study protocol (Figures 2,3).

If a myopic progression of ≥0.50 D in SE is confirmed in either eye at any scheduled follow-up visit, participants in the treatment group and control group will be dispensed new spectacles with an updated prescription to ensure best-corrected VA of 1.0 (decimal) or better.

#### 2.5.1 Primary outcome

The primary outcomes are the mean AL values measured at baseline and at each 3-month follow-up visit, from which changes will be derived for each group separately.

#### 2.5.2 Secondary outcomes

The secondary outcomes are the mean SE values measured at baseline and at each 3-month follow-up visit, from which changes will be derived for each group separately.

The secondary outcomes also include Binocular Visual Function Parameters (Negative relative accommodation and Positive relative accommodation, Accommodative response. Near point of accommodation, Accommodative Facility, Convergence-Divergence facility).

#### 2.5.3 Exploratory outcome

Adaptation and visual quality questionnaire responses may serve as potential confounding factors in myopia development. Visual quality and adaptability of Children Wearing Myopia Control spectacle lenses will also be explored. Changes in parameters over the 12-month study period will be evaluated as exploratory outcomes.

#### 2.5.4 Adverse event

Definitions and Severity Grading of Adverse Events.

Our safety monitoring protocol is based on established standards from prior clinical trials of myopia-control devices and the applicable regulatory framework for medical devices.

An Adverse Event (AE) is defined as any untoward medical occurrence, unintended disease or injury, or any untoward clinical sign (including an abnormal laboratory finding) in subjects, whether or not considered related to the investigational spectacle lenses. In our ocular health assessments, we focused on recording all ocular-related AEs.

The severity of AEs is graded according to the following standardized criteria, which have been successfully implemented in large-scale retrospective and prospective studies of myopia-control devices:

Mild: An event that is tolerable for the subject, does not affect the treatment regimen, requires no special intervention, and does not affect the subject's general health.

Moderate: An event that is intolerable for the subject, requires special intervention, and has a direct effect on the subject's {Citation} health.

Severe: This category is reserved for Serious Adverse Events (SAEs), as defined below.

Definition and Reporting of Serious Adverse Events (SAEs).

SAEs is defined as any event that is life-threatening, causes permanent harm to body function or injury to body structure, or requires medical measures to avoid the aforementioned permanent harm or injury.

All SAEs will be reported to the Principal Investigator and the relevant Ethics Committee within 24 h of the study team becoming aware of the event.

The most anticipated adverse events related to spectacle wear are peripheral blurred vision, dizziness, and eyestrain. All adverse events will be documented by study staff throughout the trial, and any reported signs or symptoms will be managed promptly.

#### 2.5.5 Criteria for study withdrawal

Participants will be withdrawn from the study for any of the following reasons:

Withdrawal of Consent: The subject or their parent/guardian requests to discontinue participation.

Protocol Violation: Significant non-compliance with the study protocol, such as failing to wear the assigned spectacles for the required minimum of 12 h per day, or initiating other myopia control treatments (e.g., atropine, contact lenses).

Safety Reason: The occurrence of SAEs, in the clinical judgment of the investigator, makes continued participation unsafe for the subject.

#### 2.5.6 Follow-up schedule

In addition to the baseline visit, scheduled follow-up examinations will occur at 3, 6, 9, and 12 months (Figure 3). Participants in both groups will also undergo additional visits at 1 week and 1 month for assessment of adaptation and visual quality.

### 2.6 Eye examinations

At baseline and at each 3-month follow-up visit, all participants will undergo ophthalmic examination, including measurements of uncorrected and corrected VA; external eye examination; ocular alignment and extraocular muscle motility; AL (MYAH; Topcon), keratometry (KR-800; Topcon), and cycloplegic and non-cycloplegic refraction (VT-10; Topcon); as well as dilated fundus examination and binocular vision function assessment.

Ocular alignment will be assessed using the cover–uncover test, and extraocular muscle motility will be evaluated through pursuit and saccadic eye movements. Intraocular pressure (IOP) will be measured with non-contact tonometry (NT-510; NIDEK), with three consecutive readings obtained and the mean value used for analysis.

#### 2.6.1 Assessment of external eye and anterior segment

Slit-lamp biomicroscopy will be used to assess the conjunctiva, cornea, anterior chamber, pupil, iris, lens, and anterior vitreous. Children presenting with acute keratoconjunctivitis at the time of examination will be excluded from cycloplegic refraction, ocular biometry, and fundus examination until resolution of the condition.

#### 2.6.2 Dilated fundus examination

Dilated fundus examination will be performed using binocular ophthalmoscopy to evaluate the vitreous, optic disc, and posterior pole after pupil dilation.

#### 2.6.3 AL examination

AL (MYAH; Topcon) was measured following the manufacturer's standard operating procedures. For each eye, three consecutive measurements were obtained during each session. Only measurements meeting the device's signal quality criteria (SNR ≥2) and demonstrating stable fixation were accepted. Quality control was performed in real time by reviewing the device's internal quality metrics and ensuring artifact-free signal traces. The acceptable inter-measurement range was defined as ≤0.06 mm based on published repeatability limits for pediatric AL measurements (TRT = 2.77 × Sw, Sw ≤ 0.02 mm). If the range of three measurements exceeded this limit, an additional measurement was taken, and the most consistent three values (within the acceptable range) were averaged for analysis. The within-subject standard deviation (Sw), repeatability limit (TRT), and coefficient of variation (CoV) were calculated to assess measurement reliability in our cohort.

#### 2.6.4 Assessment of binocular vision

binocular vision function is tested by Refractive Vision Function Tester（VED-O-3-300 TianJin China). Negative relative accommodation and Positive relative accommodation, Accommodative Response. Near point of accommodation, Accommodative Facility, Convergence-Divergence facility are all included.

#### 2.6.5 Pupil dilation and cycloplegic autorefraction

Four drops of topical cycloplegic agent (0.5% tropicamide and 0.5% phenylephrine eye drops; zhuo bi an, xingqi, China) will be administered at 5-minute intervals. At least 20 min after the final drop, pupillary light reflex and pupil diameter will be assessed. Cycloplegia will be considered adequate when the pupil shows no constriction to penlight stimulation and measures >6 mm in diameter; otherwise, an additional drop of tropicamide will be given.

Participants will be instructed to keep their eyes closed throughout the procedure, with refraction completed within 30–40 min after instillation of the final drop.

All comprehensive optometry examinations will be performed by an experienced optometrist following the standard operating procedures. The examination will include retinoscopy, corneal topography, slit-lamp biomicroscopy, and fundus examination under non-cycloplegic conditions.

If the pupil still responds to penlight stimulation, one additional drop of cycloplegic agent will be administered, and the participant will be asked to wait an additional 10 min before cycloplegic autorefraction is performed using a table-mounted autorefractor (KR-800; Topcon, Tokyo, Japan).

Refractive error will be determined from at least three consecutive readings per participant. To support data quality and myopia control monitoring, a refractive development profile will be established. The optometrists and data management team will jointly review the database to identify and correct any missing or inaccurate entries in a timely manner.

To minimize data discrepancies, all measurements will be performed using the same set of equipment, and all instruments will be calibrated before each examination session.

### 2.7 Questionnaire survey and compliance assessment

Tolerability and treatment adherence will be assessed at baseline and at each 3-month follow-up visit. To support adherence to the study protocol, all participants will be advised to maintain at least 2 h of daily outdoor time and to comply with the scheduled follow-up schedule throughout the trial. In addition, all participants will complete adaptation and visual quality questionnaires at 1 week and 1 month after lens dispensing.

To explore potential factors associated with myopia control efficacy, a customized Visual Quality and Adaptability Questionnaire will be administered to all participants (or their parents/guardians) at 1 week and 1 month after lens fitting. The questionnaire comprises five sections with 20 items, covering the following domains: visual clarity and stability; dynamic vision and spatial perception; peripheral vision and visual interference; comfort and physical adaptability; and daily functioning and overall acceptance. Each item is rated on a 5-point Likert scale indicating degree of agreement, and respondents will be instructed to select the single most appropriate response for each question. The research findings from the expert team indicated that the scale items comprehensively reflect the core dimensions of visual quality and adaptability associated with children wearing corrective spectacle lenses for myopia prevention. The Cronbach's *α* coefficient is 0.933.

### 2.8 Sample size calculation

This study is a prospective, non-randomized, active-controlled, equivalence trial. Participants will be assigned in a 1:1 ratio to receive either DMMCL group or RCDL group according to their own preferences, or those of their guardians. The equivalence margin for the primary outcome (AL change over 12 months) was set at ±0.15 mm, based on the established efficacy difference between DOT and single-vision lenses reported in the CATHAY study (−0.26 mm for axial length at 12 months). An equivalence margin of ±0.15 mm ensures that the DOT lens retains at least 55% of the clinically meaningful effect observed against single-vision lenses, while accommodating the inherent variability across studies and populations.

Based on previously reported randomized clinical trials, the 1-year mean changes in cycloplegic spherical equivalent (SE) were–0.17 D for the DIMS group (6) and −0.23 D for the DOT group (12), while the corresponding changes in AL were 0.11 mm and 0.18 mm, respectively. The AL parameter was selected for sample size calculation, using a standard deviation (SD) of 0.20 mm, derived from the observed variability in AL changes. An equivalence limit of 0.15 mm was set, reflecting the range of axial length modifications reported with various myopia control spectacle lens designs (8, 11, 20).

Sample size was calculated using PASS 15.0 (NCSS, Kaysville, UT, USA) for an equivalence trial comparing two means with a 1:1 allocation ratio. Assuming 80% power, a one-sided significance level of 0.05, a true difference of 0.05 mm (12), and a standard deviation of 0.20 mm, the estimated sample size was 102 participants (51 per group). With an anticipated 15% dropout rate over the 12-month follow-up period, the final sample size was adjusted to 120 participants (60 per group).

### 2.9 Statistical analysis

A study database will be constructed using Microsoft Excel (Redmond, WA, USA). For primary analyses, data from the right eye of each participant will be used to determine refractive status and ocular parameters. Continuous variables will be summarized as mean (SD), and categorical variables as frequencies and percentages. Between-group differences in refractive error, demographic characteristics, and questionnaire responses will be evaluated using the independent Student's *t*-test (or Mann-Whitney *U*-test for non-normally distributed data). Statistical analyses will be performed using IBM SPSS Statistics 20.0 (IBM Corp., Armonk, NY, USA), with a two-sided significance level set at *p* < 0.05. Data analysis also will be performed using R software (version 4.2.2; R Foundation for Statistical Computing; http://www.Rproject. org), and Free Statistics software (version 2.1; Beijing Free Clinical Medical Technology Co., Ltd.) was used for the analysis.

Baseline demographic and clinical characteristics will be summarized descriptively using mean (standard deviation) or median (interquartile range) for continuous variables, and frequency (percentage) for categorical variables, separately for the experimental and control groups. Standardized mean differences (SMD) will be calculated to quantify the magnitude of between-group differences at baseline, with SMD < 0.1 indicating adequate balance, 0.1–0.3 indicating modest imbalance, and >0.3 indicating meaningful imbalance requiring additional adjustment.

We will plan propensity score matching as the primary analytical approach to mitigate selection bias. We will apply 1:1 nearest-neighbor matching without replacement, using a caliper of 0.2 times the standard deviation of the logit-transformed propensity score. This matching procedure will yield matched pairs with adequate covariate balance, as indicated by standardized mean differences (SMD) < 0.1 for all included covariates. This approach effectively approximates a pseudo-randomized comparison within the matched cohort, reducing the risk of confounding by measured covariates. Subgroup analyses and E-value analysis will also to assess robustness to unmeasured confounding.

Statistical analyses will be performed in as-allocated population, defined as all participants who were initially assigned to either the experimental or the control group according to participant or guardian preference, sensitivity analyses conducted on the per-protocol (pp) population. For the primary outcome (change in AL at 12 months), the two one-sided tests (TOST) procedure will be applied to test for equivalence between the two treatment groups at a significance level of *α* = 0.05. The 90% confidence interval for the between-group difference will be calculated, and equivalence will be declared if the entire confidence interval falls within the prespecified equivalence margin of ±0.15 mm.

To handle missing data in the repeated measures, generalized estimating equations (GEE) with an unstructured correlation matrix will be employed. The GEE models will include time, treatment assignment, and their interaction as fixed effects, with adjustment for prespecified confounders including age, sex, baseline SE, and number of myopic parents.

The primary analysis of equivalence will be performed on the matched cohort using the two one-sided tests (TOST) procedure at the two-sided *α* = 0.05 level. Equivalence will be concluded if the 90% confidence interval for the between-group difference in the primary outcome falls entirely within the pre-specified equivalence interval (−Δ, +Δ). A supportive repeated-measures analysis will be performed using generalized estimating equations (GEE) with an exchangeable working correlation structure on the matched cohort, incorporating all follow-up measurements as repeated outcomes and including the group-by-time interaction term. This analysis is intended to assess whether the equivalence conclusion is sustained over time and whether the temporal trajectory of the outcome differs between groups. It is considered supportive, not confirmatory, for the primary equivalence conclusion. To assess robustness to residual confounding, two sensitivity analyses will be conducted on the full (unmatched) cohort: (1) multivariable linear or logistic regression adjusting for all pre-specified covariates plus the propensity score as a continuous covariate; and (2) inverse probability of treatment weighting (IPTW) using the estimated propensity scores with stabilized weights. The consistency of the effect estimates across these sensitivity analyses with the primary analysis will be assessed to evaluate the robustness of the equivalence conclusion.

The primary equivalence analysis is conducted on the propensity-score-matched cohort. As a result, unmatched participants who may differ systematically from the matched sample are excluded from the primary inferential analysis. This may limit the generalizability of the anticipated findings to the broader population. The as-allocated cohort is used only for descriptive summaries, and all inferential conclusions are drawn from the matched sample. Sensitivity analyses on the full cohort using multivariable adjustment and inverse probability of treatment weighting will be conducted to assess the robustness of the findings to the exclusion of unmatched participants.

A per-protocol (PP) analysis will be performed as a supplementary analysis on the subset of matched participants who completed the intervention without major protocol deviations. The PP analysis will use the same TOST procedure on the matched PP subset. This analysis is intended to assess whether protocol deviations influence the equivalence conclusion and is interpreted as a sensitivity check.

Changes in binocular visual function parametersthese parameters will be calculated as the difference between baseline and the 12-month follow-up visit. Within-group comparisons will be performed using paired *t*-tests to evaluate the significance of these changes over the study period.

Exploratory outcomes include between-group comparisons of adaptability and visual quality, as assessed by the questionnaire. Independent *t*-tests will be used to compare the two groups in terms of changes in questionnaire scores from 1 week and 1 month.

### 2.10 Data management and quality assurance

The study will use standardized and structured Case Report Forms. Two data entry clerks will independently perform double data entry and conduct consistency checks. All data modifications must be documented in an audit trail, recording the modifier, modification time, original value, and reason for the modification. Clinical Research Associates will conduct sampling and comparison of 20% of the source data according to the schedule, focusing on the consistency of informed consent forms, inclusion/exclusion criteria, primary efficacy endpoints, and serious adverse events with the CRF records.

## 3 Discussion

The prevention and control of childhood myopia has remained a longstanding public health challenge, a situation further exacerbated by the widespread use of digital screen-based devices (22). Multiple myopia control methods are employed in myopia prevention and management. Spectacle lenses, as a non-invasive and readily accepted tool for myopia control, are widely favored by children, adolescents, and their parents or guardians (18).

A range of clinical interventions are currently available for myopia control, including spectacle lenses, contact lenses, and combined pharmacological-optical approaches (23). Regardless of the treatment strategy employed, the most significant therapeutic objective is to decelerate the progression of myopia following its onset (24). Several publications currently indicate that myopic defocus mechanism lenses or RCDL retards eye growth and myopia progression (5, 16, 25, 26). DOT myopia control spectacle lenses as RCDL are based on contrast theory. DOT lenses contain thousands of light-scattering elements, which serve to reduce the retinal image contrast (27). Animal studies have consistently demonstrated that inducing myopic defocus in the peripheral retina can effectively slow axial elongation (28). In addition, several clinical trials have shown that increasing the peripheral addition power of myopia control lenses based myopic defocus mechanism helps to inhibit axial length growth (2, 6, 18).

However, no clinical trial has directly compared the efficacy of DMMCL and RCDL within the same cohort. The primary objective of this study is to evaluate whether DMMCL are equivalent to RCDL in controlling myopia progression, as measured by AL elongation over 12 months. This study will also preliminarily discuss two distinct mechanisms of visual signal intervention to determine whether different visual stimuli can prevent visual response fatigue and sensory adaptation, thereby yielding more effective signals for inhibiting axial length growth.

The primary equivalence analysis is conducted on the propensity-score-matched cohort. As a result, unmatched participants who may differ systematically from the matched sample are excluded from the primary inferential analysis. This may limit the generalizability of the anticipated findings to the broader population. The as-allocated cohort is used only for descriptive summaries, and all inferential conclusions are drawn from the matched sample. Sensitivity analyses on the full cohort using multivariable adjustment and inverse probability of treatment weighting will be conducted to assess the robustness of the findings to the exclusion of unmatched participants.

In conclusion, this study will provide evidence to supple the current literature by investigating the impact of refractive-correcting lenses utilizing both defocus and retinal contrast modulation mechanisms on changes in myopic refractive error. Specifically, this study will evaluate the efficacy of DMMCL in delaying myopia progression in school-aged children; analyze the trends in refractive error and axial length changes in myopic adolescents wearing spectacle lenses with different optical designs; and preliminarily explore the potential mechanisms underlying the efficacy of the dual-mechanism design. The results of this trial, when available, will inform clinical practice by clarifying whether DMMCL offer a viable alternative to RCDL for myopia control. Future studies with longer follow-up and multicenter designs will be needed to confirm the generalizability of the anticipated findings.

Given the single-center and non-randomized nature of this study, any future findings should be interpreted as preliminary real-world evidence. If equivalence is demonstrated, the anticipated results will support efficacy of the RCDL spectacle lens in this population. The anticipated results should not be interpreted as sufficient to single-handedly establish a basis for policy change. Larger, multi-center randomized controlled trials are warranted to confirm these observations and to more robustly inform future clinical practice guidelines and pediatric eye-care policy. In the future, wearable devices could be utilized to better standardize or objectively record various types of time data, enabling us to investigate how daily activities at different times affect myopia prevention and control in children who wear spectacle, as well as to assess the impact of these confounding factors on the results.

### 3.1 Rationale for the study design

The selection of an appropriate control group is critical for evaluating the efficacy of clinical trials. While previous myopia control studies have often used placebo or single-vision (SV) lenses as controls, the present study employs RCDL as the sole comparator to enable a direct comparison of myopia control effects between RCDL and DMMCL in children aged 6–12 years. The rationale for not including a placebo or SV control group is as follows:

1. SV lenses have demonstrated minimal impact on myopia progression (29).
2. Once myopic children are identified, appropriate treatment strategies must be selected to manage myopia progression; therefore, establishing a placebo control is impractical.
3. In recent clinical practice, obtaining informed consent from children and their parents solely for the use of single-vision (SV) lenses in a myopia control study has become increasingly challenging, as parents are generally aware of the availability of specialized myopia control lenses and may be reluctant to accept a non-intervention or placebo control.
4. According to the International Myopia Institute (IMI) consensus (23, 30, 31) and several meta-analyses, single-vision (SV) lenses are not recommended as a first-line intervention for myopia control. In contrast, myopia control spectacle lenses, which represent the least invasive option, offer a favorable safety profile for long-term use.

In view of the above considerations and the risk–benefit profile of participants in long-term clinical trials, the present study will employ RCDL—an intervention with established myopia control efficacy—as the comparator.

## 4 Supporting information

S1 File. SPIRIT-outcomes 2025 checklist. (PDF). S2 File. Questionnaires on adaptation and visual quality. (PDF)

### 4.1 Trial status

At the time of manuscript submission, participant recruitment had commenced. The study protocol (version 2.0, dated 1 August 2026) was used. A protocol amendment (version 2.0, dated 1 August 2026) was introduced after recruitment had commenced. The amendments included: (1) clarification of the hierarchical analytical framework for the primary equivalence analysis; (2) standardization of data collection procedures. The first participant was enrolled on 20 January 2026, and recruitment is expected to be completed by 15 December 2026. Trial registry update: pending ethics approval. The trial registry record (https://www.chictr.org.cn/ as chiCTR2600116438) will be updated to reflect the protocol amendment within 30 calendar days of receiving formal ethics committee approval. Confirmation that outcome data were not reviewed before the amendment. We wish to emphasize that the amendment was finalized and submitted before any outcome data were reviewed or analyzed. All changes were based on operational considerations and baseline data review only.
