# Supplementary material for: Control effect of dual-mechanism myopia control lenses on myopia in children and adolescents: a prospective, 12-month, non-randomized, parallel-controlled, and single-center trial protocol
Source: Front Med (Lausanne). 2026 Sep 10;13:1931165. doi: 10.3389/fmed.2026.1931165 (PMC13600819; doi:10.3389/fmed.2026.1931165)
Supplement: Supplementary file 1 [file Datasheet1.pdf]

questionnaires on adaptation and visual quality

5 points: Always (>80% of the time)
4 points: Often (approximately 50-80% of the time)
3 points: Sometimes (approximately 20-50% of the time)
2 points: Rarely (<20% of the time)
1 point: Never

Part One: Visual Clarity and Stability

1. When viewing distant objects (e.g., blackboards, road signs), vision feels clear and effortless.

(1) Never — (2) Rarely — (3) Sometimes — (4) Often — (5) Always

2. When viewing close objects (e.g., books, phones), vision feels clear and effortless.

(1) Never — (2) Rarely — (3) Sometimes — (4) Often — (5) Always

3. Vision remains clear in slightly dim environments (e.g., dusk, overcast days).

(1) Never — (2) Rarely — (3) Sometimes — (4) Often — (5) Always

4. Vision feels stable without fluctuating between clear and blurry.

(1) Never — (2) Rarely — (3) Sometimes — (4) Often — (5) Always

Part Two: Dynamic Vision and Spatial Perception

5. When walking or climbing stairs, can accurately judge the height and distance of steps.

(1) Never — (2) Rarely — (3) Sometimes — (4) Often — (5) Always

6. During physical activities (e.g., running, playing ball), can clearly see moving objects.

(1) Never — (2) Rarely — (3) Sometimes — (4) Often — (5) Always

7. When shifting focus suddenly from distant to near objects (or vice versa), can adjust focus quickly without noticeable delay.

(1) Never — (2) Rarely — (3) Sometimes — (4) Often — (5) Always

8. Accurate judgment of an object's three-dimensionality (distance, depth), e.g., during leisure activities or when doing detailed crafts.

(1) Never — (2) Rarely — (3) Sometimes — (4) Often — (5) Always

Part Three: Peripheral Vision and Visual Distractions

9. When turning your eyes to see objects at the sides, do you experience clear vision?

(1) Never — (2) Rarely — (3) Sometimes — (4) Often — (5) Always

10. Do you notice halos, glare, or rainbow-like streaks around the edges of your glasses (especially when looking at lights)?

(1) Always — (2) Often — (3) Sometimes — (4) Rarely — (5) Never

(This question is reverse-scored)

11. Do you perceive slight "shadows" or "double vision" in your field of view?

(1) Always — (2) Often — (3) Sometimes — (4) Rarely — (5) Never

(This question is reverse-scored)

12. When turning your head, do you notice slight shaking or distortion of objects seen through your lenses?

(1) Always — (2) Often — (3) Sometimes — (4) Rarely — (5) Never

(This question is reverse-scored)

Part Four: Comfort and Physical Adaptability

13. When wearing glasses, the bridge of the nose and ears feel comfortable without noticeable pressure or pain.

(1) Never — (2) Rarely — (3) Sometimes — (4) Often — (5) Always

14. After prolonged wear, no sensations of eye soreness, swelling, or fatigue occur.

(1) Never — (2) Rarely — (3) Sometimes — (4) Often — (5) Always

15. After wearing glasses for extended periods, you do not experience headaches or dizziness.

(1) Never — (2) Rarely — (3) Sometimes — (4) Often — (5) Always

16. Do you feel your glasses are heavy and often have the urge to take them off?

(1) Always — (2) Often — (3) Sometimes — (4) Rarely — (5) Never

(This question is reverse-scored)

Part Five: Daily Functionality and Overall Acceptance

17. After reading or doing homework for more than 30 minutes, you still feel relaxed.

(1) Never — (2) Rarely — (3) Sometimes — (4) Often — (5) Always

18. When using electronic devices (such as tablets or computers), you feel visual comfort.

(1) Never — (2) Rarely — (3) Sometimes — (4) Often — (5) Always

19. You (or your child) feel willing and accustomed to wearing these glasses all day.

(1) Never — (2) Rarely — (3) Sometimes — (4) Often — (5) Always

20. Overall, you (or your child) are satisfied with the visual performance of these myopia control lenses.

(1) Never — (2) Rarely — (3) Sometimes — (4) Often — (5) Alway
